# Effect of Photosensitiser Chlorin E6 on Cancerous Bone Tumor Cells Using Photodynamic Therapy

**DOI:** 10.32604/or.2025.071919

**Published:** 2026-02-24

**Authors:** Frank Traub, Muhammad A. Panezai, Michaela Moisch, Julia Melke, Leonard Schöbel, Tilmann Busse, Fei Xing, Jiachen Sun, Ulrike Ritz

**Affiliations:** 1 Department of Orthopaedics and Traumatology, Biomatics Group, Institute for Immunology and Research Center for Immunotherapy (FZI), University Medical Center of the Johannes Gutenberg University, Langenbeckstr. 1, Mainz, 55131, Germany; 2 Department of Pediatric Surgery, West China Hospital, Sichuan University, Chengdu, 610041, China

**Keywords:** Photodynamic therapy, photosensitizer, Chlorin e6, bone cancer, bone metastasis, Cluster of Differentiation 164

## Abstract

**Objectives:**

Photodynamic therapy (PDT) is a minimally invasive method used in the treatment of various cancers and skin diseases, but it is not widely used in bone cancer, where the current therapy is often not effective and accompanied by side effects. Alternative and more effective therapies like PDT are needed. In this *in-vitro* study, the effect of the photosensitizer (PS) chlorin e6 (Ce6) on cancerous bone tumor cells using PDT was examined.

**Methods:**

A total of 27 tissue specimens from patients with primary bone cancers or bone metastases of different origins were genetically characterized and treated with PDT. Following a 24-h incubation, cell viability was determined, and the effect of PDT on cell migration was analyzed over 48 h.

**Results:**

We could demonstrate that the effect on proliferation of PDT in combination with the PS Ce6 was best in cells isolated from primary osteosarcoma and in bone metastases from mammary carcinomas. Besides proliferation, PDT was also effective in inhibiting the migration of these cells. A statistically significant correlation between the PDT effect and CD164 gene expression was detected, indicating that a high expression of this gene could result in a higher effectiveness of the photodynamic treatment.

**Conclusion:**

This study analyzes for the first time the effect of PDT in bone cancers and metastases and shows the potential of treating these cancer types with Ce6 PDT.

## 1 Introduction

Bone cancer, including primary tumors such as osteosarcoma and chondrosarcoma, as well as bone metastases commonly arising from breast, prostate, and lung cancer, remains a significant challenge in medicine. Despite significant advances in oncology and cancer research, standard therapies, such as surgical resection and chemotherapy, have largely remained unchanged over the past 30 years. These treatments are often accompanied by severe side effects that significantly impact patients’ mental and physical quality of life. Furthermore, some tumors are therapy-resistant and persist in the affected area or metastasize early. This underscores the urgent need for new, more effective, and less invasive therapeutic options [1].

Photodynamic therapy (PDT) is a promising cancer treatment method that harnesses light. The term “photodynamic”, first used by Hermann von Tappeiner in 1904, describes a photochemical process in which a photosensitizer (PS) absorbs light, subsequently generating reactive oxygen species (ROS), inducing cell death [2]. This effect in PDT is triggered by apoptotic, necrotic, or autophagic mechanisms [3,4]. Since the photosensitizer is activated only by exposure of the targeted surface to light of a specific wavelength, PDT is considered minimally invasive for patients [5]. This is highly relevant for treating tumors in complex regions, as preserving the surrounding sensitive bone and soft tissue is crucial for maintaining function and patient quality of life.

The first application of PDT in oncology was described in the 1970s [6]. Since that time, PDT has been shown to exert therapeutic benefits in the treatment of multiple cancer types. Photosensitizers are absorbed by tumor cells and accumulate within them. Upon light exposure, these non-toxic PS produce ROS, leading to the ablation of tumor cells. A key advantage of PDT is that healthy cells and surrounding tissue take up less PS and are consequently less sensitive to PDT [7]. Overall, PDT is an effective, safe, and affordable treatment option for tumors and their metastases.

PDT has shown promise for treating osteosarcoma, a highly aggressive bone tumor, by overcoming challenges such as chemotherapy resistance and local recurrence. Innovative delivery systems that combine PDT with other therapies can enhance therapeutic effects, leading to improved patient outcomes [8]. Several methods exist for delivering light in PDT, but fiber-optic technology is considered optimal due to its ability to precisely target tissues, provide uniform light distribution, and treat both superficial and deep-seated lesions [9].

*In vitro* studies have demonstrated that PDT can effectively target metastatic bone lesions, with varying responses observed among different cancer cell lines [10]. A clinical trial indicated that PDT, when combined with vertebroplasty, is safe and can significantly reduce pain in patients with vertebral metastases [11]. Various PS have been tested in *in vitro* studies in different osteosarcomas or bone metastases, including 5-Aolevulinic acid (5-Ala) [10], Methylene Blue [12], Purpurin 18 [13], and others [2]. However, further PS testing is required to identify the optimal treatment for osteosarcoma and bone metastases.

One particularly promising photosensitizer is Chlorin e6 (Ce6), a second-generation photosensitizer used in PDT. Ce6, a natural chlorophyll derivative, is a key component of Fotolon, a clinically applied photosensitizer that has been used in approximately 20,000 patients since 2001 [14]. Compared to first-generation photosensitizers, chlorin e6 causes virtually no photosensitivity. It is excited at a higher wavelength (660–670 nm) than Photofrin (630 nm), enabling the treatment of tumors located deeper within the tissue [15,16], and is Food and Drug Administration (FDA) and European Medicines Agency (EMA) approved. There is evidence of its therapeutic effects on adenocarcinoma and breast neoplasms [17] as well as in squamous cell carcinoma, where Ce6 triggers PANoptosis and enhances antitumor immunity [18]. The exact mechanisms of action and which signaling pathways are affected are still not completely clarified. 5-Ala PDT induces pyroptosis via the JNK signaling pathway, whereas Ce6 seems to influence the Akt/mTOR pathway [19].

The objective of this study was to investigate the effect of the photosensitizer chlorin e6 (Ce6) on cancerous bone tumor cells using PDT. For this purpose, a collection of tumor tissues from 27 patients with various primary cancerous conditions was examined. The effect of the PDT treatment with different concentrations of the photosensitizer chlorin e6 was analyzed to ascertain the lowest concentration of the photosensitizer at which the full effect of the photodynamic treatment can be observed. Migration assays were performed to test whether treatment of cells with Ce6 had an effect on the migration potential of tumor/metastasis cells. The expression of different genes known to influence cell adherence, cell proliferation, and growth was assessed in the tumor cell samples to determine whether they influence the efficacy of PDT. The genes tested were CD138 (syndecan-1, SDC-1), CD146 (MCAM, melanoma cell adhesion molecule), CD164 (endolyn), and GD2 (ganglioside), as well as GAPDH (glyceraldehyde-3-phosphate dehydrogenase) as a housekeeping gene. CD138 has been used as a prognostic factor in various cancers for several years. High expression of syndecan-1 is a negative prognostic factor in some cancers and correlates with poor response to therapy [20]. Similar aspects apply to CD146. Previous studies have reported its expression in diverse cancers, and a correlation has been detected between increased expression of this molecule and metastatic capacities of tumors. Results of various studies suggest that CD146 plays a major role in tumor development as well as in angiogenesis [21]. Moreover, it seems to be a potential marker to predict metastatic potential and might be used as a therapeutic target [22]. In different human cancers, CD164 has been reported to play an important role in the maintenance and progression [23]. GD2 (disialoganglioside-2) is highly expressed by various cancers like neuroblastomas, melanomas and retinoblastomas, Ewing sarcomas, osteosarcomas, soft tissue sarcomas, etc. [24].

## 2 Materials and Methods

### 2.1 Cell Lines, Sample Collection and Treatment

The cell line Saos-2 (HTB-85, ATCC, Manassas, VA, USA) was used as a control cell line. The STR profile was provided by ATCC (D5S818, D13S317, D7S820, D16S539, CSF1PO, vWA, TH01, TPOX) + Amelogenin, and the absence of mycoplasma contamination was analyzed and confirmed with the VenorGeM OneStep Kit (11-8025, Minerva Biolabs GmbH, Berlin, Germany).

Primary osteoblasts were isolated and characterized as described before [25]. Absence of mycoplasma contamination was analyzed and confirmed with the VenorGeM OneStep Kit (11-8025, Minerva Biolabs GmbH, Berlin, Germany).

The tissue samples were collected during various surgical procedures in the Department of Orthopedic and Trauma Surgery at the University Medical Center, Johannes Gutenberg University (JGU) in Mainz. The ethics committee of the Landesärztekammer Rhineland-Palantine approved the project (2024-17697). The study was conducted in accordance with the Declaration of Helsinki. Informed consent was obtained from all participants, and consent was also obtained from Department of Orthopaedics and Traumatology, University Medical Center of the Johannes Gutenberg University, Mainz, Germany. All patients, whose tissue samples were used in this research, agreed to the collection, storage, and scientific use of their tissues. All the available tissue samples were diagnosed as malignant. The tissue defined as tumor tissue was removed intra-operatively and subsequently processed as described below. The amount of harvested tissue depended on tumor size and therefore varied accordingly. Tissue types are listed in Table 1.

**Table 1:** Overview of tissues used for cell isolation with age and sex (f—female, m—male) and type of metastasis or primary tumor. Cells were isolated from 27 different tissues. BM, bone metastasis, followed by the primary tumor; PC, primary cancer

Age, Sex	Diagnosis
68, f	BM—breast cancer
48, f	BM—breast cancer
73, m	BM—breast cancer
72, m	BM—prostate cancer
85, f	BM—thyroid cancer
82, m	BM—soft tissue sarcoma
51, f	BM—soft tissue sarcoma
24, f	BM—soft tissue sarcoma
66, f	BM—soft tissue sarcoma
75, f	BM—soft tissue sarcoma
34, f	BM—soft tissue sarcoma
48, m	BM—squamous cell cancer
60, m	BM—lung cancer
68, f	BM—lung cancer
57, f	BM—lung cancer
56, m	BM—lung cancer
63, m	BM—kidney cancer
44, f	BM—rectum carcinoma
74, m	BM—kidney carcinoma
73, m	BM—urothelial cancer
49, m	PC—Osteosarcoma
52, f	PC—Osteosarcoma
35, m	PC—Osteosarcoma
69, f	PC—Chondrosarcoma
56, m	PC—Chondrosarcoma
56, m	PC—Chondrosarcoma
79, m	PC—Chondrosarcoma

### 2.2 Treatment of the Tissue Samples

**Figure 1:**
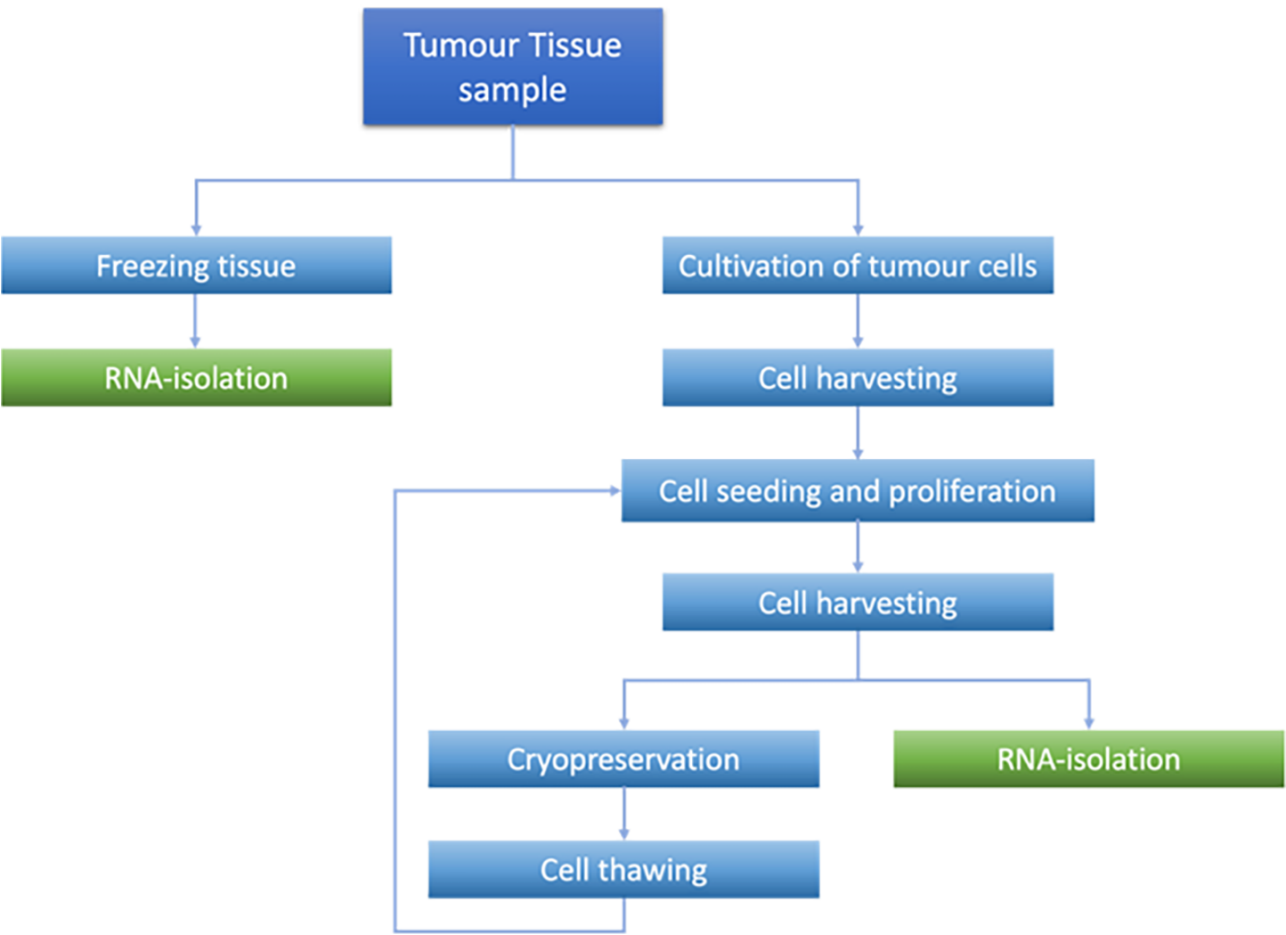
Processing steps of tumor tissue and cells

Tissue samples were collected from surgery and treated as schematically shown in Fig. 1. The tissue was from different sources, as shown in Table 1.

The tumor tissue was minced into small fragments and placed in a 6-well flat-bottom cell culture plate. Each well was filled with 3 mL of growth medium composed of Dulbecco’s Modified Eagle’s Medium (DMEM, Thermo Fisher Scientific, Darmstadt, Germany) supplemented with 10% fetal bovine serum (FBS) and 1% penicillin-streptomycin (P/S, Sigma Aldrich, Darmstadt, Germany). The plate was then incubated at 37°C in a humidified atmosphere containing 5% CO_2_. Cell outgrowth was monitored microscopically (Evos XL Digital Imaging Device, Fisher Scientific, Frankfurt, Germany), and cells were passaged upon reaching approximately 90% confluence. Quality control criteria included successful initial outgrowth and sustained proliferation after the first passage. Cultures that failed to proliferate or did not survive the initial passage were excluded from further analyses.

### 2.3 RNA isolation and PCR

In this study, RNA was isolated from two sources of the same origin, namely tumor tissue and tumor cultured cells, using Precellys Lysing Kit (peqLab #91-PCS-CK28, VWR, Darmstadt, Germany) for tumor tissue and peqGOLD MicroSpin Total RNA Kit (12-6831-02, VWR, Darmstadt, Germany) for outgrowth cells, respectively.

From Tumor Tissue

Tumor tissue was preserved in RNAlater (Sigma Aldrich, Taufkirchen, Germany), an aqueous, non-toxic RNA stabilization reagent, and stored at −80°C until further processing. Approximately 20 mg of tumor tissue was lysed and homogenized for RNA isolation using the Precellys Tissue RNA Kit (KT03961-1-003.2, Bertin Technologies, Berlin, Germany). Total RNA was isolated and purified with RNA-binding columns according to the kit protocol.

From Outgrown Cells

RNA from 100,000 cultured tumor cells was extracted using the peqGOLD MicroSpin Total RNA Kit (12-6831-02, peqLab Biotechnology GmbH, Erlangen, Germany) following the manufacturer’s instructions. During isolation, DNase treatment was performed according to the manufacturer’s protocol.

A total of 1 μg of isolated RNA was reverse transcribed into complementary DNA (cDNA) using a reaction mix containing dNTPs (4you4 dNTPs Mix, BIORON GmbH, Ludwigshafen, Germany), Random Primers (Promega, Madison, WI, USA), and M-MuLV Reverse Transcriptase (New England Biolabs, Ipswich, MA, USA) according to the respective manufacturer’s protocols.

For gene expression analysis, PCR amplification was conducted using SYBR Green dye (PowerUp™ SYBR^®®^ Green Master Mix, Applied Biosystems, Foster City, CA, USA) and sequence-specific primers (listed in Table 2). GAPDH was employed as the reference gene to normalize expression levels. Amplification was performed on a qTower3 thermocycler (Jena Analytik, Jena, Germany) with the following cycling conditions: an initial activation step at 95°C for 2 min, followed by 40 cycles of denaturation at 95°C for 15 s, and annealing/extension at 60°C for 25 s. PCR data were analysed by calculating dCt values, normalizing target gene expression to the internal housekeeping gene GAPDH for relative quantification. Correlation between the effect of the PDT treatment and the expression of the analyzed target genes was measured using the Pearson correlation coefficient (Pearson’s r).

**Table 2:** Primer sequences, listed in the order 5^′^-3^′^

Primer name	Sequence forward primer	Sequence reverse primer
GAPDH	CGA CCA CTT TGT CAA GCT CA	AGG GGA GAT TCA TGT TGG TG
GD2	AGA GCT CCC GTG AGT GGT TA	ATC CCG CAC AAG AGT GCT AG
CD138	AGC TGA AAG GCC GGG AAC	CGC TCT CTA CTG CCG GAT TC
CD146	CCG TCT CGT AAG AGC GAA	CAG GGA AGG GAG CTG AAG TG
CD164	CCA GTG CCA ACA GCC AAT TC	ATT ACA GCC TGC ACA CCC AA

### 2.4 Photodynamic Therapy (PDT) Treatment

Isolated cells were assessed for their response to PDT. A total of 25,000 cells were seeded into a 24-well flat, clear-bottom black plate (ibidi, Gräfelfing, Germany) and incubated for 24 h at 37°C with 5% CO_2_.

After the incubation period, cells were treated with the photosensitizer Ce6 (Santa Cruz Biotechnology, Heidelberg, Germany, CAS 19660-77-6). All subsequent experimental steps were conducted in the dark to prevent premature activation of the photosensitizer. Ce6 working solutions were prepared at a concentration of 1.0 µg/mL (1.67 µm) from a 3.75 mg/mL stock solution (in phosphate-buffered saline [PBS, Sigma Aldrich, Merck, Darmstadt, Germany, D8537, pH 7.0]/5% DMSO, stored at −20°C) in serum-free DMEM (1% P/S). This concentration was the lowest effective dose, tested in preliminary experiments with concentrations ranging from 0.1 to 3 µg/mL (0.16–5.02 µm). A negative control (without Ce6) was included. Preliminary experiments demonstrated that treatment with light had no effect on the cells. For each treatment, 1 mL of the respective Ce6 solution was added to the wells, and the cells were incubated for 2 h. This time point was chosen due to existing literature [26,27] and preliminary experiments testing different time points. After 2 h, the medium was aspirated, and after one washing step with PBS, fresh medium was added to the cells. Afterward, half of the wells were irradiated using a PDT apparatus with a 665 nm red light applicator at a total laser output of 230 mw (Richard Wolf GmbH, Knittlingen, Germany), resulting in an irradiance of 8.33 mW/cm^2^ at the center of the well and a fluence of 0.5 J/cm^2^. The irradiance was determined in preliminary experiments using a Thorlabs S120VC power sensor connected to a Thorlabs PM100A power meter (Thorlabs, Lübeck, Germany). The applicator was positioned vertically over each well without contacting the medium, and the distance between the light source and the bottom of the well was set so that the light cone fully encompassed (illuminated) the entire well. Each well was exposed to light for 60 s. Pre-experiments confirmed that laser irradiation under these conditions did not significantly heat the medium, ensuring that thermal effects did not influence the results.

After light exposure, the medium in all wells (both irradiated and non-irradiated controls) was replaced with fresh growth medium containing DMEM (5% FBS, 1% P/S). The cells were returned to the incubator and allowed to recover overnight.

### 2.5 Cell Viability Measurement (alamarBlue Assay)

The alamarBlue cell viability assay (Merck, Darmstadt, Germany) was used to assess the effects of PDT treatment on tumor cells at 24 and 48 h post-treatment.

To measure cell viability, the alamarBlue reagent was diluted at a ratio of 1:10 in DMEM supplemented with 5% FBS and 1% P/S. A total of 500 µL of the diluted alamarBlue solution was added to each well of the treated cells. The plates were then incubated for 4 h at 37°C in a humidified atmosphere with 5% CO_2_.

Following incubation, four 100 µL aliquots from each well were transferred into a 96-well plate. Fluorescence intensity was measured using the GloMax^®®^ Multi-Detection System (Promega, Walldorf, Germany) with excitation at 545 nm and emission at 590 nm to quantify cell viability.

### 2.6 Migration Assay

The migration assay was conducted using ibidi 2-well culture inserts (ibidi, Gräfelfing, Germany) in a 24-well plate. A total of 3 × 10^4^ cells were seeded into each chamber of the 2-well culture insert and incubated for 24 h to allow for cell adhesion. After the incubation period, the culture insert was carefully removed, creating a defined cell-free gap, and PDT treatment was performed as previously described. Standardization of the migration assay was reached by using 2-well-culture inserts to create a standardized gap, always the same cell number in all experiments, the same incubation times and cell media, as well as identical microscopic parameters, including an automated image analysis. Cell migration was monitored over 3 days, using the Celloger Mini Cell-Imaging System (HB Instruments, Tübingen, Germany). Cell migration was assessed by monitoring the rate at which cells invaded the defined gap and by quantifying the migrated area using the software included in the cell imaging system (HB Instruments, Tübingen, Germany).

### 2.7 Statistics

All experiments were performed at least three times, with measurements carried out in triplicate. Statistical analyses were conducted using GraphPad Prism 10 (GraphPad Software Inc., Boston, MA, USA), and results are presented as medians and quartiles.

For normally distributed data, a one-way analysis of variance (ANOVA) was performed. Depending on the outcome of Levene’s test for equality of variances, pairwise comparisons were conducted using either Tukey’s HSD or Games–Howell post hoc tests. The Kruskal–Wallis test was applied for non-normally distributed data, followed by a Bonferroni-corrected Conover–Iman analysis. Pairwise comparisons for non-parametric data were conducted using the Mann–Whitney U test.

Statistical significance was set at *p* < 0.05 and reported as follows: *p < 0.05, **p < 0.01, ***p < 0.005, and ****p < 0.001. To account for multiple tests, *p*-values were adjusted using the Bonferroni–Holm method.

## 3 Results

### 3.1 Gene Expression Analyses

Gene expression of four selected target genes was analyzed using reverse transcription qPCR.

**Figure 2:**
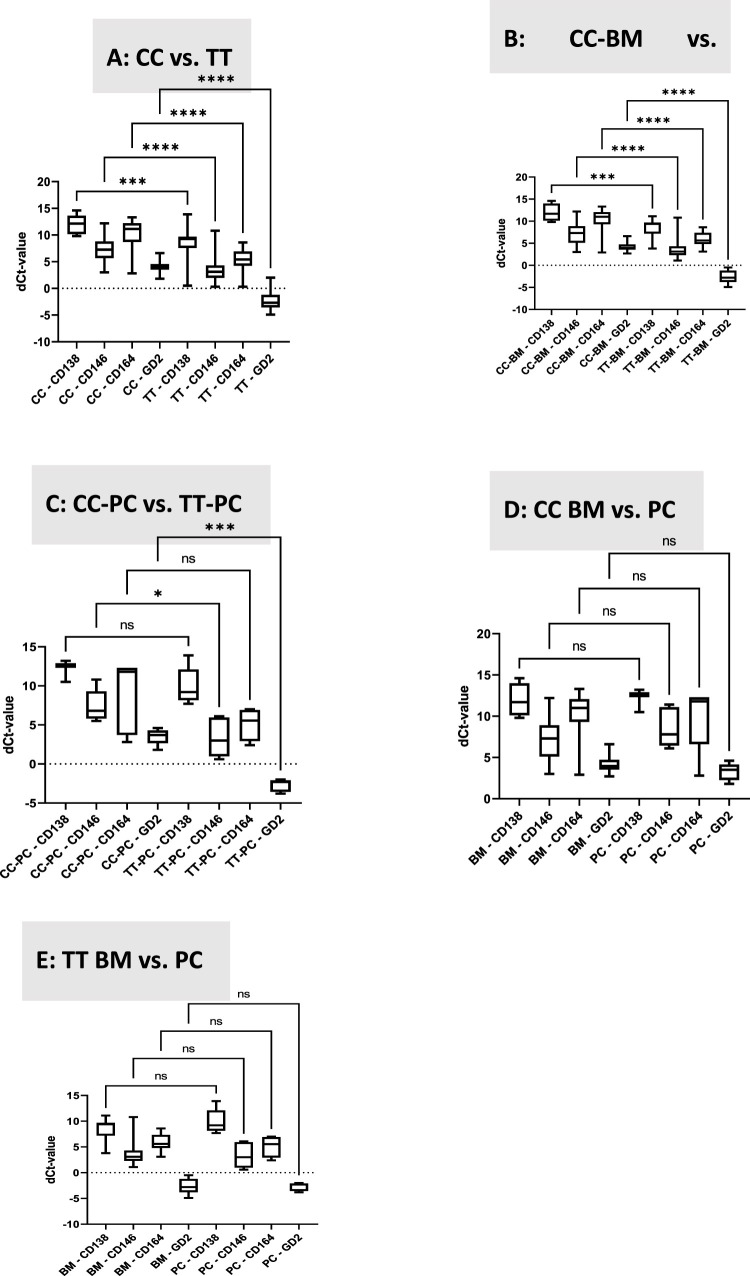
Transcript levels of CD138, CD146, CD164, and GD2. dCt levels are presented, and gene expression was normalized relative to the housekeeping gene GAPDH. **A**. Comparison between gene expression of the isolated cells after cell culture (CC) and the original tumor tissue (TT). **B**. Comparison between gene expression of the isolated cells after cell culture from bone metastases (BM-CC) and the original bone metastase tissue (BM-TT). **C**. Comparison between gene expression of the isolated cells after cell culture from primary cancers (CC-PC) and the original primary cancer tissue (TT-PC), **D**. Comparison between gene expression of the isolated cells after cell culture from bone metastases (CC-BM) and cell cultures from primary cancers (CC-PC). **E**. Comparison between gene expression of the tissues from bone metastases (TT-BM) and tissues from primary cancers (TT-PC). CC, cell culture; TT, tumor tissue; BM, bone metastasis; PC, primary cancer. *p < 0.05, ***p < 0.001, ****p < 0.0001, ns: not significant

Gene expression was determined in the tissue (TT) as well as in the cells in culture (CC) to characterize the differences that arise after some time of culture. Fig. 2A shows the comparison between dCt-values from cells in culture with the corresponding bone tumor tissue. Interestingly, all genes are significantly higher expressed in the tumor tissue than in the corresponding cells after some time of culture, and this pattern was observed for all probes collectively (Fig. 2A) as well as when looking at primary bone cancer tumors (PC, Fig. 2B) and bone metastases (BM, Fig. 2C) separately. Examining expression of the genes in the cell culture and tumor tissue, respectively, and comparing the expression in primary bone cancer tumors to bone metastases, no significant differences could be detected (Fig. 2D and E).

Overall, the most highly expressed target gene was the disialoganglioside gene (GD2). In most tissue samples, its expression was greater than that of the housekeeping gene (GAPDH). CD146, melanoma cell adhesion molecule (MCAM), was the gene with the second-highest expression, whereas CD164 demonstrated lower, and CD138 the lowest expression.

The high expression of the GD2 gene in the examined samples confirms previous research showing that GD2-targeting T-cells can control the growth of osteosarcoma [28]. Moreover, GD2 is discussed as a therapeutic target in antibody-mediated therapy in osteosarcoma [29]. Similarly, the relatively high expression of the CD146 gene in the samples confirms previous research showing the role of the CD146 gene in the progression of osteosarcoma [30]. Interestingly, it has also been shown that the expression of CD146 in osteosarcoma cell lines is higher compared to normal osteoblast-like cells [31]. High expression of CD146 is also associated with metastasis and a poor outcome in osteosarcoma [32], and can be used as a therapeutic target to reduce the occurrence of bone metastases [33]. The low expression of CD138 was expected and used as a negative marker control, as it has been described that it is rarely expressed in sarcomas and metastases [34]. The role of CD164 is not well defined—it’s high expression has been described for example in Ewing sarcoma [35].

### 3.2 Cell Viability after PDT Treatment

Cell viability was measured 24 and 48 h after treatment with chlorin e6 (Ce6) and PDT. The optimal concentration of chlorin e6 (Ce6) was determined experimentally. The results showed that the lowest, most efficient, and effective Ce6 concentration was 1.0 µg/mL. In a first step, the optimal time point for measuring viability was evaluated and determined to be 48 h. The fluorescence intensity measured after treatment with alamarBlue assay is directly proportional to the cell viability in the samples and was set in relation to the cells not treated with PDT.

**Figure 3:**
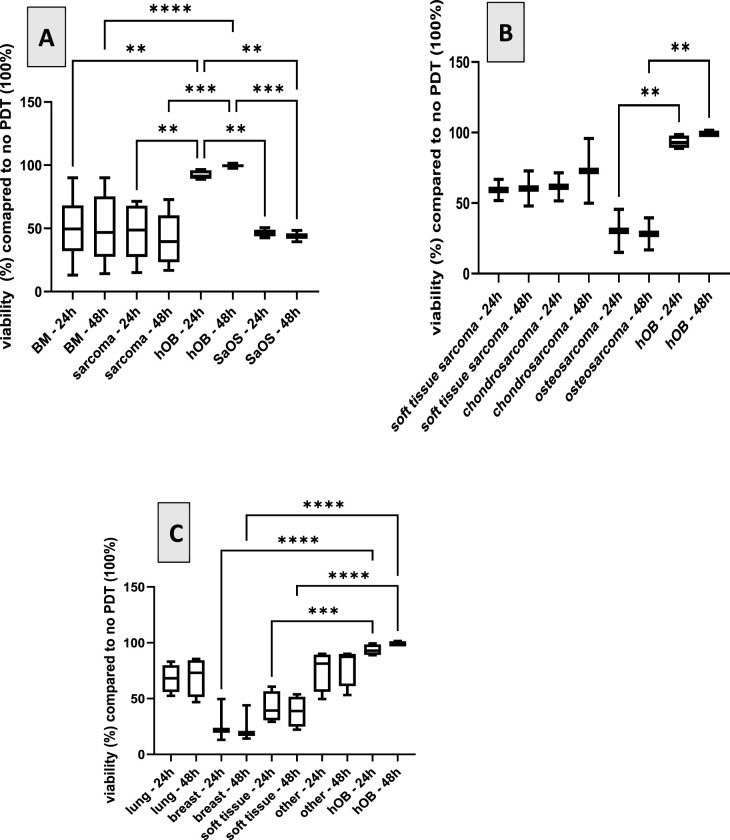
Viability in % of primary cells isolated from different carcinogenic tissues 24 h and 48 h after PDT compared to cells not treated with PDT (100%). **A**. Effect of PDT on the two controls (primary osteoblasts (hOB)) and osteosarcoma cell line SaOS-2 compared to bone metastases (BM) and bone sarcomas. **B**. Effect of PDT on primary bone tumors: osteosarcoma, chondro- and soft tissue sarcoma. **C**. Effect of PDT on bone metastases with distinct origins, origin indicated in the *x*-axis. Viability is compared to non-treated cells (100%). **p < 0.01; ***p < 0.001, ****p < 0.0001

Fig. 3 shows the viability of the different cells after treatment with PDT compared to the control, not treated with PDT, respectively. Human primary osteoblasts were tested as a negative control, as it was expected that these cells would not take up the PS Ce6, and PDT should show no effects. Although it has not yet been demonstrated specifically for osteoblasts, several studies have shown that non-cancerous cells take up markedly less chlorin e6 (Ce6) and, consequently, do not exhibit a PDT effect [2,36]. These findings were used as the rationale for employing osteoblasts as a negative-control in the present study. As an additional control, the osteosarcoma cell line SaOS-2 was also included. Fig. 3A shows the expected effect of PDT on the two controls. Whereas no effect on viability was observed with the osteoblasts, the viability of SaOS-2 was reduced to less than 50%. Moreover, we compared the effects of PDT with Ce6 on bone metastases to bone sarcomas without differentiating between the exact phenotypes. In all cases, PDT reduced the viability of the cells beneath 50% compared to the group with no treatment. Splitting the results in regard to the type of tumor, Fig. 3B demonstrates that on primary bone tumors, the effect of PDT is most pronounced in osteosarcoma compared to chondro- and soft tissue sarcoma. Regarding bone metastases with distinct origin the effect of PDT is most pronounced in bone metastases originating from breast carcinoma followed by soft tissue carcinoma. The effect of PDT is least pronounced in bone metastases originating from lung cancer.

Only few studies exist analyzing the effect of PDT in osteosarcoma. Some studies used 5-Ala as PS, showing an effect [2,10,37], but hardly any literature utilizes Ce6. Most studies regarding the effects of Ce6-PDT in osteosarcomas were performed with cell lines and not with primary cells isolated from the primary tumor or bone metastases. One study showed an effect of Ce6-PDT in a murine melanoma model [38]. Yu et al. combined Ce6 with deoxy-d-glucose and showed a prolonged mice survival compared to non-treated groups [39]. Our study deals with the effect of Ce6 combined with PDT on different sarcomas and metastases in the bone region. We observed differences among the cells derived from different sources: in the case of sarcoma, the best effect is seen with osteosarcoma, followed by soft tissue and chondrosarcoma. Regarding metastases, the strongest effect is observed in metastases originating from primary breast carcinoma, followed by metastases from soft tissue tumors. A significantly less effect was observed in bone metastases originating from other primary tumors like lung or other, e.g., prostate cancer. To our knowledge, this is the first study analyzing the effect of the PS Ce6 used in PDT in primary cells analyzed from primary bone tumors or bone metastases. One can conclude that different effects are observed depending on the source of the tumor. This should be investigated in future projects using a larger cohort of tumors.

### 3.3 Correlation between Gene Expression and PDT Effectiveness

**Figure 4:**
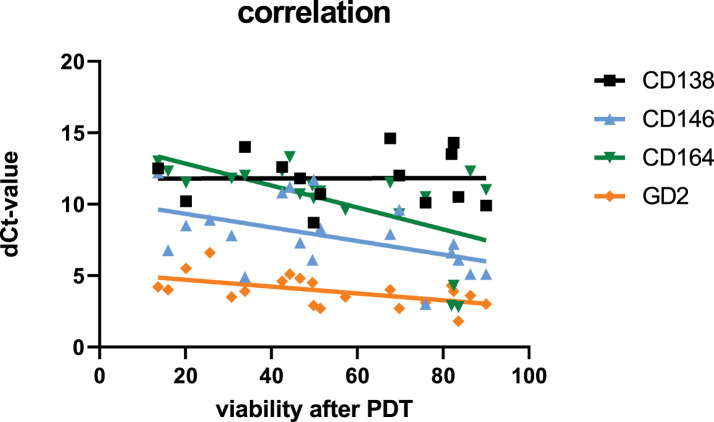
Correlation between gene expression of the tested genes (dCt values of CD138, CD146, CD164, and GD2) with viability of the tested cells after PDT treatment (48 h)

Correlation between the effect of the PDT treatment and the expression of the analyzed target genes was measured using the Pearson correlation coefficient (Pearson’s r) and the corresponding *p*-value. No correlation was observed between viability and expression of CD138. However, a correlation was detected regarding the other testes genes CD146, CD164, and GD2: the higher the dCt-value, the lower the viability of the cells after PDT—meaning the higher the expression of the respective genes, the lower the PDT effect (Fig. 4). A significant correlation was only detected for CD164. Table 3 shows the values:

**Table 3:** Pearson correlation coefficient and *p*-value of correlation data (dCt-gene expression vs. viability after 48 h)

Gene	r	*p*
CD138	−0.005	0.965
CD146	−0.485	0.032
CD164	−0.576	0.005
GD2	−0.561	0.019

Predicting the effects of PDT in tumor treatment is increasingly feasible through various methodologies that assess key factors influencing treatment outcomes. Recent studies highlight the importance of monitoring oxygenation levels, dosimetry, and the interplay of photosensitizers, light, and oxygen in predicting therapeutic efficacy [40]. Predicting the effect of PDT in tumor treatment using gene expression levels is not common. Some research indicates that genes are differentially expressed following PDT [41], and some deal with predictive gene expression models for responses to various anticancer therapies, among others, also PDT [42]. We chose four typical tumor markers to correlate the effect of PDT with gene expression in the tumor. We could demonstrate a correlation that might help predict the effectiveness, but it has to be further analyzed in detail. One approach that should be pursued in follow-up studies to confirm the correlation between PDT efficacy and CD164 gene expression is to perform experiments using CD164 inhibitors and antagonists, as well as siRNA-mediated knock-down, to assess cell proliferation and migration.

While gene expression profiling shows potential for predicting PDT responses, it is essential to consider the inherent tumor heterogeneity and the complexity of gene interactions, which may complicate predictions. Further research is needed to refine these predictive models and validate their clinical applicability.

### 3.4 PDT Shows an Effect on Cell Migration

**Figure 5:**
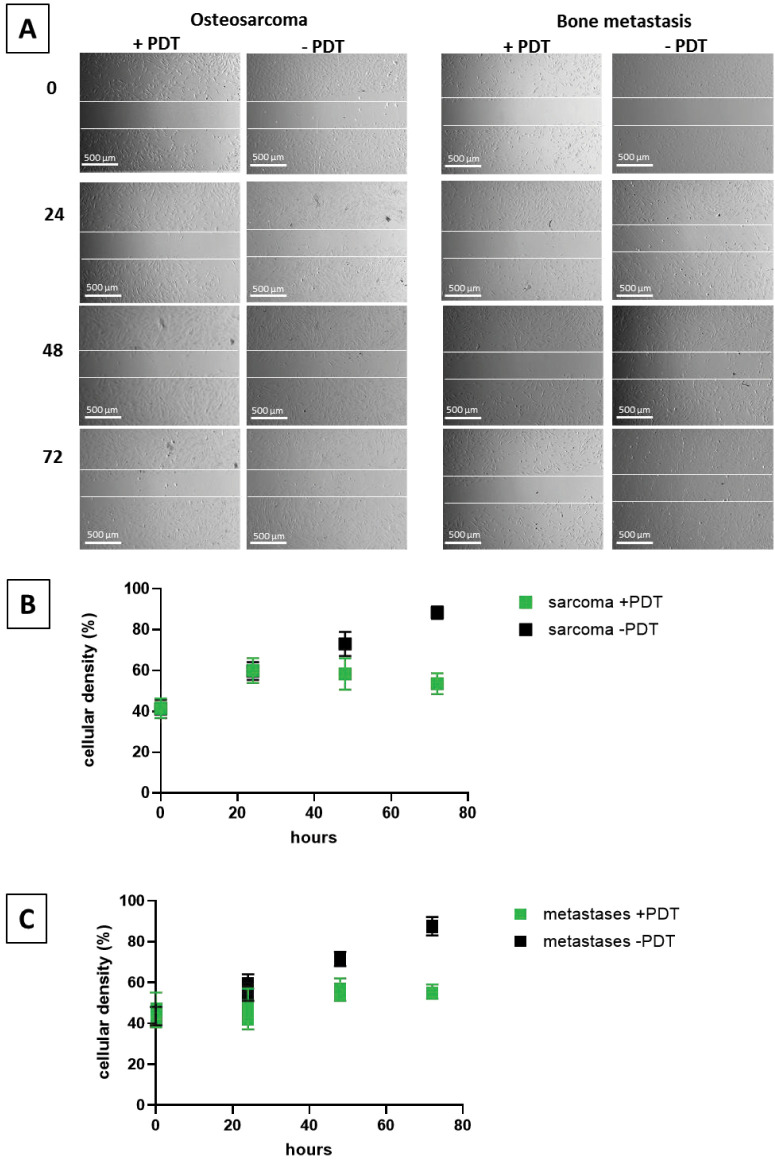
Migration assay. Example of cell migration of cells isolated from an osteo sarcoma and a bone metastasis from a breast-CA after 0, 24 h, 48 h, and 72 h (**A**) and quantification of cell density in sarcoma (**B**) and metastases (**C**) with and without PDT. **A**: Scale bar indicates 500 µm, and the white demarcation lines indicate the initial wound boundaries

In order to define the effect of PDT on the migration of the tumor cells, migration assays were performed. Cells were seeded with cell culture inserts that created a defined gap in 24 wells. After removal of the plastic, PDT was performed, and the growth of cells was observed via a microscope, and the cellular density was quantified. Fig. 5A shows an example of metastatic and sarcoma cells treated with and without PDT directly and 24, 48, and 72 h after seeding, respectively. After 24 h, no difference is observed between the treated and untreated cells, but after 48 and 72 h, the gap is still visible in the cells treated with PDT, whereas in the cells without treatment, migration of the cells into the gap is observed. These exemplary qualitative results are confirmed by quantification of cellular density in the observed area: Only marginal differences are observed after 24 h, but after 48 as well as after 72 h, a statistically significant difference in cellular density (*p* ≤ 0.05) was detected between the treated and the untreated groups (Fig. 5B,C).

## 4 Discussion

Our study provides compelling evidence that chlorin e6-mediated photodynamic therapy (Ce6-PDT) holds substantial promise for treating primary bone tumors and metastases. In contrast to prior studies conducted primarily in osteosarcoma cell lines such as HOS, 143B, and MG-63 [43–45], we employed patient-derived primary cells to capture tumor heterogeneity and metastatic potential. This strategy enhances the translational relevance of our findings, addressing the persistent gap in preclinical validation for Ce6-PDT in bone oncology [46]. Ce6 was specifically selected for this study due to its activation by longer wavelengths (660–670 nm), facilitating deeper tissue penetration—a critical property for treating deeply embedded bone tumors, particularly during intraoperative procedures [44]. Ce6 is also favored due to its favorable pharmacologic profile, which includes potent antitumor and anti-inflammatory activity, strong ROS generation, a large absorption coefficient, minimal tissue residue, and a well-documented safety record [47].

A primary objective was to assess the impact of Ce6-PDT on cell viability. Compared to literature, our used power density was 0.5 J/cm^2^, relatively low [16]. In contrast to our study, in the literature, established cell lines are used, which might be more resistant to PDT compared to our primary cells utilized. As we observe effects with the low power density, it seems to be sufficient. Our findings demonstrated that PDT, in combination with Ce6, significantly reduced the viability of most tested cells to below 50% compared to untreated control groups. This effect was most pronounced in cells isolated from primary osteosarcoma and in bone metastases originating from breast carcinomas. Conversely, the least pronounced effect of PDT was observed in bone metastases derived from lung cancer. These results highlight the heterogeneity of bone metastases and suggest that the primary tumor’s origin significantly influences PDT responsiveness.

Ce6-PDT also demonstrated effective inhibition of migration in primary bone tumor and metastasis cells. This crucial anti-metastatic effect was qualitatively and quantitatively demonstrated through wound healing assays. The persistence of a defined cell-free gap in the treated cell cultures after 48 and 72 h, in contrast to the observed closure of the gap in untreated control cells, unequivocally indicates reduced cellular motility. This finding is further supported by statistically significant differences in cellular density within the observed area. The wound healing assay is a widely accepted and cost-effective method for assessing cancer cell motility and invasiveness, making it a reliable surrogate for metastatic potential. The time span for observing statistical effects varies in the literature from 24 to 72 h [48–50]. Observed effects after 72 h are less often documented than after 24 h, yet, in contrast to the mentioned studies with short time incubation times, established cell lines were tested, not primary cells, as in our study. This might explain the differences as primary cells grow more slowly than immortalized cells [51]. The observed inhibition of cell migration by Ce6-PDT is consistent with a growing body of literature on PDT’s anti-migratory and anti-metastatic capabilities across various cancer types, including esophageal and breast carcinomas. Mechanistically, this effect often results from PDT-induced apoptosis and the disruption of cytoskeletal integrity or extracellular matrix remodeling. Our previous research using 5-aminolevulinic acid (5-ALA)-mediated PDT in breast cancer-derived bone metastatic cell lines showed decreased migration alongside increased apoptosis [10]. Recent studies have also demonstrated Ce6-PDT’s ability to induce pyroptosis—a pro-inflammatory form of programmed cell death—which not only contributes to tumor cell killing but may also alter the tumor microenvironment to suppress metastatic dissemination [52]. Furthermore, nanoparticle-supported PDT has shown significant improvements in therapeutic efficacy, including reduced metastasis [53]. This is particularly relevant given the limitations of conventional photosensitizer delivery in dense and hypoxic tumor tissues like bone. The dual demonstration of reduced cell viability and migration in our study suggests that Ce6-PDT exerts a multifaceted anti-tumor effect. While loss of viability naturally contributes to reduced motility, the preservation of a cell-free area and decreased cellular density after PDT strongly implies that Ce6-PDT directly interferes with cellular mechanisms underlying migration. This dual action enhances PDT’s therapeutic value, targeting both tumor mass and its invasive potential—a key driver of cancer-related mortality [54]. Additionally, the anti-migratory effects of PDT may be intricately linked to its ability to stimulate anti-tumor immune responses [55]. If PDT induces immunogenic cell death, including pyroptosis, it might trigger the release of DAMPs (damage-associated molecular patterns) [56], which in turn recruit immune effector cells and reshape the tumor microenvironment. This immunomodulation may directly or indirectly contribute to the inhibition of tumor cell migration. Understanding this complex interplay will be crucial for optimizing PDT protocols and designing combination strategies that amplify both anti-proliferative and anti-metastatic effects.

A pivotal component of this study involved analyzing gene expression profiles and their correlation with PDT efficacy. CD164, a transmembrane sialomucin, showed a positive correlation with treatment response—tumors with higher CD164 expression exhibited significantly lower viability following Ce6-PDT. While CD164 is generally linked to aggressive tumor phenotypes and drug resistance [23], our findings suggest that it may sensitize cells to ROS-mediated damage, possibly due to altered redox regulation or enhanced photosensitizer uptake. Moreover, our data suggest that this marker might identify cells sensitive to PDT and could serve as positive predictive biomarkers for Ce6-PDT.

CD146 (MCAM) is frequently implicated in tumor progression, angiogenesis, and metastasis, and is linked with poor prognosis and therapeutic resistance in several cancer types [57]. GD2, a ganglioside highly expressed in aggressive tumors including osteosarcomas and breast cancers, is known to promote proliferation and metastasis, often via c-Met and mTOR pathways [58]. However, our data show no statistically significant correlation of gene expression and PDT.

The gene expression correlation points toward a future where PDT could be personalized based on tumor molecular profiling. Patients with high CD164 expression might be prioritized for PDT. Building on these findings, future validation will proceed through advanced *ex vivo* and *in vivo* models. Three-dimensional culture systems and organoids derived from patient tumors will provide a more physiologically relevant environment to confirm these gene-treatment associations. Intermediate *in vivo* platforms like the chick chorio-allantoic membrane (CAM) model offer valuable insights into tumor-PDT dynamics under vascularized conditions. Successful validation in these systems will justify translation to more comprehensive animal models and, ultimately, human trials.

Combination strategies may further enhance Ce6-PDT effectiveness, especially against resistant tumor phenotypes. PDT has shown promise when used alongside chemotherapeutics, immune checkpoint inhibitors, or photothermal therapies. These synergies may help counteract the limitations imposed by tumor hypoxia or survival signaling pathways active in CD146- and GD2-positive cells [4]. Rationally designed combination therapies tailored to a tumor’s molecular profile may represent the next frontier in PDT-based treatment.

In summary, our study offers critical insights into the application of Ce6-PDT in bone oncology. By using patient-derived cells and linking gene expression to treatment outcomes, we lay the groundwork for predictive biomarker development and personalized therapy design. The promising *in vitro* results, especially for osteosarcoma and breast cancer-derived metastases, warrant further preclinical development using integrated model systems that mirror the complexities of human disease.

***Limitations***

Despite its promise, Ce6-PDT has inherent limitations. The limited penetration depth of light in tissues remains a key barrier to treating deep-seated malignancies effectively [59]. Treatment efficacy is further influenced by factors such as photosensitizer distribution, light source characteristics, tissue optical properties, and tumor heterogeneity [4]. Uneven Ce6 penetration and heterogeneous light exposure may result in incomplete tumor ablation. Furthermore, our study’s limited sample size and gene panel restrict the generalizability of findings. Thus, broader gene profiling and optimized delivery strategies are essential.

## 5 Conclusions

This study investigates the potential of Ce6-mediated photodynamic therapy (Ce6-PDT) for treating bone cancers and metastases, demonstrating encouraging effects in reducing cell viability and tumor migration. It identifies key gene expression correlations, where elevated CD164 levels enhance PDT efficacy, while increased expression of CD146 and GD2 diminish it, suggesting these markers could inform personalized treatment strategies. Despite promising findings, limitations such as a small sample size, a restricted gene panel, and the *in vitro* nature of the study highlight the need for further validation. Future research should expand genetic profiling, address delivery and tissue-specific challenges of PDT in bone, and explore synergistic combination therapies. To facilitate clinical translation, subsequent studies will validate results using advanced organoid and 3D culture systems, progressing to intermediate *in vivo* models like the chick chorio-allantoic membrane (CAM) model, before advancing to comprehensive animal studies. Elucidating the molecular mechanisms and developing advanced delivery platforms will be essential for optimizing Ce6-PDT’s therapeutic potential.

## Data Availability

The data presented in this study are available on request from the corresponding author, [Ulrike Ritz], upon reasonable request.
